# Relationship between Xanthine Oxidoreductase Redox and Oxidative Stress among Chronic Kidney Disease Patients

**DOI:** 10.1155/2018/9714710

**Published:** 2018-07-08

**Authors:** Hiroyuki Terawaki, Tomoya Hayashi, Takayo Murase, Ryutaro Iijima, Kaito Waki, Yoshihiro Tani, Takashi Nakamura, Kazunobu Yoshimura, Shunya Uchida, Junichiro James Kazama

**Affiliations:** ^1^Department of Internal Medicine, Nephrology, Teikyo University Chiba Medical Center, Chiba, Japan; ^2^Department of Nephrology and Hypertension, Fukushima Medical University, Fukushima, Japan; ^3^Department of Physiology, Meiji University of Integrative Medicine, Kyoto, Japan; ^4^Radioisotope and Chemical Analysis Center, Sanwa Kagaku Kenkyusho Co., Ltd., Mie, Japan; ^5^Department of Internal Medicine, Nephrology, Teikyo University School of Medicine, Tokyo, Japan; ^6^Pharmacological Study Group, Pharmaceutical Research Laboratories, Sanwa Kagaku Kenkyusho Co., Ltd., Mie, Japan

## Abstract

Xanthine oxidase (XO), an isoform of xanthine oxidoreductase (XOR), is thought to increase the cardiovascular burden among chronic kidney disease (CKD) patients via oxidative radical production. Plasma XOR redox, which is characterized by the ratio of XO to total XOR, changes under different oxidative conditions associated with kidney dysfunction. However, the relationship between plasma XOR redox and oxidative stress (OS) is unclear. Thus, we aimed to clarify whether OS is related to XOR redox. We used the redox state of human serum albumin (HSA) as a marker to investigate the status of OS in CKD patients. HSA is composed of human mercaptoalbumin (HMA), which possesses not oxidized cysteine residues, reversibly oxidized human nonmercaptoalbumin-1 (HNA-1), and strongly oxidized human nonmercaptoalbumin-2 (HNA-2). The subjects included 13 nondialysis patients (7 males and 6 females) with varying degrees of kidney function. We found that ƒ(HMA) was negatively (*R* = −0.692, *P* = 0.0071) and ƒ(HNA-1) was positively (*R* = 0.703, *P* = 0.0058) correlated with plasma XO/XOR. ƒ(HNA-2) showed no correlation with XO/XOR (*R* = 0.146, *P* = 0.6412), indicating that plasma XOR redox is not related to the irreversible oxidation of HSA. In conclusion, plasma XOR redox is closely related to HSA redox, particularly reversible oxidation of HSA.

## 1. Introduction

Patients with chronic kidney disease (CKD) not only often have end-stage kidney disease but also are at risk of developing cardiovascular diseases (CVD) [1, 2]. That said, the importance of oxidative stress (OS) is emerging as a potential cause of the high CVD incidence among CKD patients. OS is highly correlated with kidney dysfunction [3], and increased OS is closely related to a high CVD incidence [4].

Xanthine oxidase (XO) may be related to increased OS among CKD patients. XO is the oxidative radical-forming isoform of xanthine oxidoreductase (XOR), which is well-known as a urate-producing enzyme. XO is formed by reversible or irreversible oxidation of another isoform, xanthine dehydrogenase (XDH). Recently, we reported that XOR redox, which is defined as the ratio of XO to total XOR (XO and XDH), changes the oxidative condition in association with kidney dysfunction [5]. Because the XOR inhibitor (so-called “XO” inhibitor) allopurinol reduces CVD risk to approximately 30% among CKD patients [6, 7], XOR redox and OS are thought to be closely related.

This study aimed at clarifying whether OS is related to XOR redox. We used the redox state of human serum albumin (HSA redox) as a marker of OS. HSA is a protein composed of 585 amino acids; the amino residue at position 34 from the N-terminus is a cysteine possessing a mercapto group (SH group). HSA is a mixture of human mercaptoalbumin (HMA) in which the mercapto group is not oxidized, human nonmercaptoalbumin-1 (HNA-1) in which the disulfide bond formation is reversibly oxidized by either cysteine or glutathione [8], and human nonmercaptoalbumin-2 (HNA-2) which is strongly oxidized and becomes sulfenic (−SOH), sulfinic (−SO_2_H), or sulfonic (−SO_3_H) [9].

We measured HSA redox concurrently with XOR redox in 13 nondialysis CKD patients with different levels of kidney function. Our results revealed a close relationship between XOR redox and OS.

## 2. Methods

### 2.1. Collection of Clinical Data and Specimens

The study was performed according to the principles of the Declaration of Helsinki and was approved by the local ethics committee (Fukushima Medical University, approval number 2349). All subjects provided written informed consent.

The study involved 13 CKD patients, 7 males and 6 females, aged 32.1–83.8 (62.1 ± 18.8) years. Patient characteristics are shown in Table 1. No patient had received antioxidant agents such as ascorbic acid or vitamin E. Patients on corticosteroid therapy and those with malignancy were excluded from this study.

Physical measurements including height, weight, and blood pressure were taken, and then blood samples were collected to measure XOR and HSA redox: from 2 mL of blood, plasma was obtained by centrifugation at 4°C immediately after blood sampling from each patient; these samples were stored at −80°C until analysis.

In addition, biochemical tests, including serum creatinine level, were measured. As a marker of renal function, the estimated glomerular filtration rate (eGFR) was calculated using the CKD-EPI equation modified for Japanese using a Japanese coefficient [10].

### 2.2. Measurement of HSA Redox

The redox state of albumin was determined by high-performance liquid chromatography (HPLC). The HPLC system consisted of an M-504F autosampler (Eicom, Kyoto, Japan; injection volume, 20 *μ*L of plasma diluted by 10-fold with 50 mM sodium phosphate, 150 mM sodium chloride buffer) and a 1525 binary pump (Waters, Milford, MA, USA) as well as Empower 2 system software (Waters). The chromatograph was obtained using a 2996 photodiode array detector (detection area, 210–400 nm with 1 nm step; Waters). A Shodex-Asahipak ES-502N 7C column (10 × 0.76 cm I.D., DEAE-form for ion-exchange HPLC; Showa Denko, Tokyo, Japan; column temperature, 35.0 ± 0.5°C) was used in this study. Linear gradient elution was performed with varying ethanol concentrations (0-1 min, 0%; 1–50 min, 0→10%; 50–55 min, 10→0%; and 55–60 min, 0%) for serum in 0.05 M sodium acetate and 0.40 M sodium sulfate mixture (pH 4.85) at a flow rate of 1.0 mL/min. Deaeration of the buffer solution was performed with an inline degasser AF (Waters).

**Figure 1:**
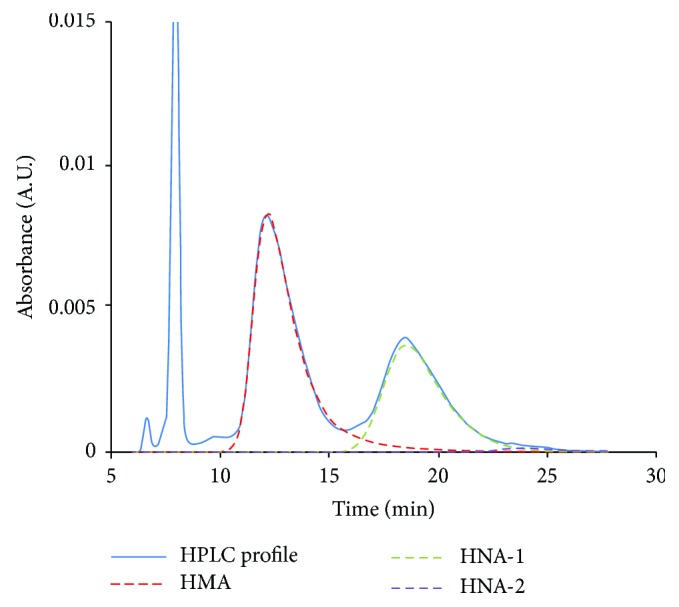
Representative HPLC profile and numerical curve fitting.

HPLC profiles obtained from these procedures were subjected to numerical curve fitting with PeakFit version 4.05 simulation software (SPSS Inc., Chicago, IL, USA), and each peak shape was approximated by a Gaussian function (Figure 1). Next, the levels for the HMA, HNA-1, and HNA-2 compared to total HSA were calculated (ƒ(HMA), ƒ(HNA-1), and ƒ(HNA-2), resp.).

### 2.3. Measurement of XOR Redox

Measurement of XOR and XO activities was performed by liquid chromatography combined with triple quadrupole mass spectrometry (LC-TQMS method). The detail of LC-TQMS method is described in a previous report [5]. Based on the XOR and XO activities obtained using the LC-TQMS method, the ratio of XO to XOR (XO/XOR) was calculated.

### 2.4. Data Analysis

Statistical analyses were performed using the statistical software StatView 9.3 (SAS Institute Inc., Cary, NC, USA) and EZR (Jichi Medical University Saitama Medical Center, Saitama, Japan), which is a graphical user interface for R (the R Foundation for Statistical Computing). EZR is a modified version of R commander (version 2.13.0, University of Vienna, Vienna, Austria) designed to add statistical functions frequently used in biostatistics [11].

To determine the magnitude of the correlation, we used Pearson's correlation coefficient (*R*). Item-category data (gender, primary disease, prescription, and past history) were introduced into the analysis as dummy variables. The correlation was considered significant when the *P* value was less than 0.05 (5%) with Fisher's *Z* transformation.

## 3. Results

Table 2 shows the clinical data. Serum creatinine levels in patients were 0.56–4.89 mg/dL, and eGFR was 8.22–99.33 mL/min/1.73 m^2^. The redox states of HSA, ƒ(HMA), ƒ(HNA-1), and ƒ(HNA-2) were 52.73–81.55%, 17.43–45.19%, and 0.99–2.21%, respectively.

**Figure 2:**
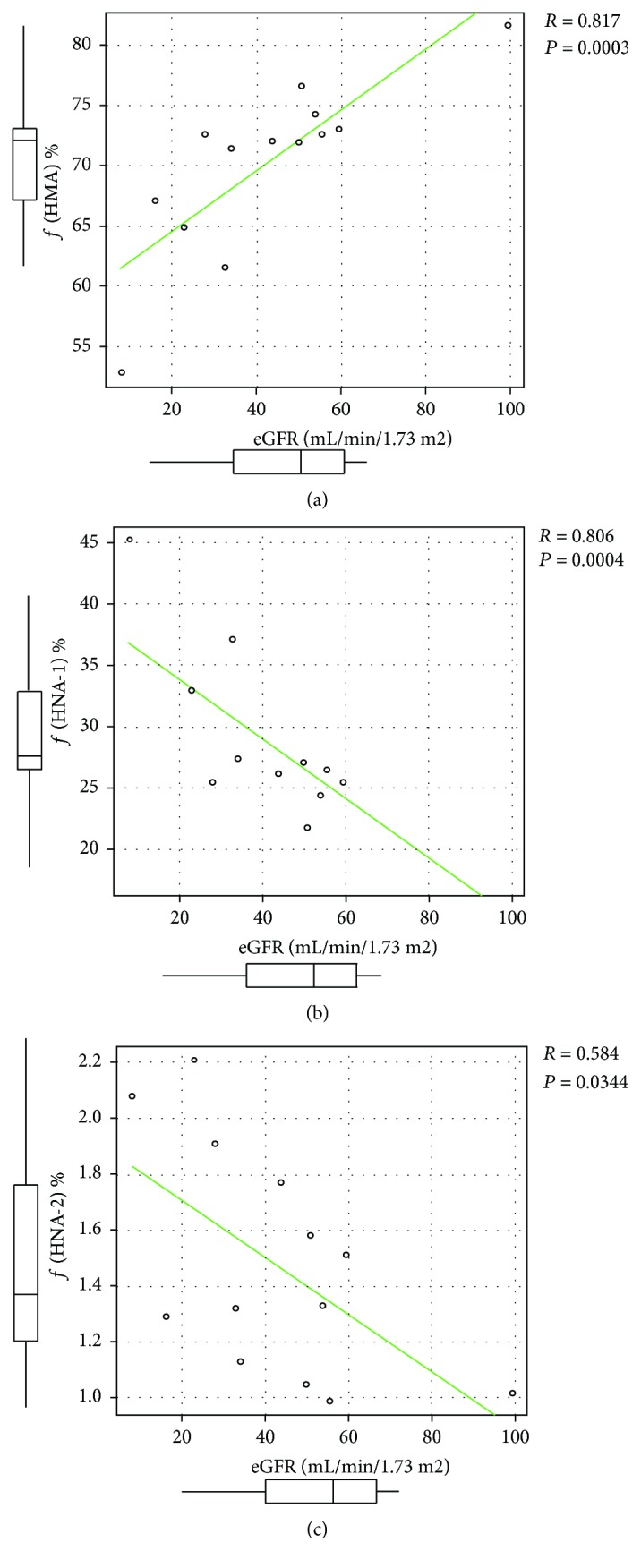
Relationship between estimated glomerular filtration ratio (eGFR) and (a) ƒ(HMA), (b) ƒ(HNA-1), and (c) ƒ(HNA-2). For each value, ƒ(HMA) shows a positive correlation, whereas ƒ(HNA-1) and ƒ(HNA-2) show a negative correlation with eGFR.

The relationship between ƒ(HMA), ƒ(HNA-1), ƒ(HNA-2), and renal function (eGFR) is shown in Figure 2. A significantly positive correlation between eGFR and ƒ(HMA) was observed (*R* = 0.817, *P* = 0.0003). In contrast, both ƒ(HNA-1) and ƒ(HNA-2) were significantly negatively correlated with eGFR (*R* = −0.806, *P* = 0.0004 and *R* = −0.584, *P* = 0.0344, resp.), indicating that decreased renal function contributes to HSA oxidation.

**Figure 3:**
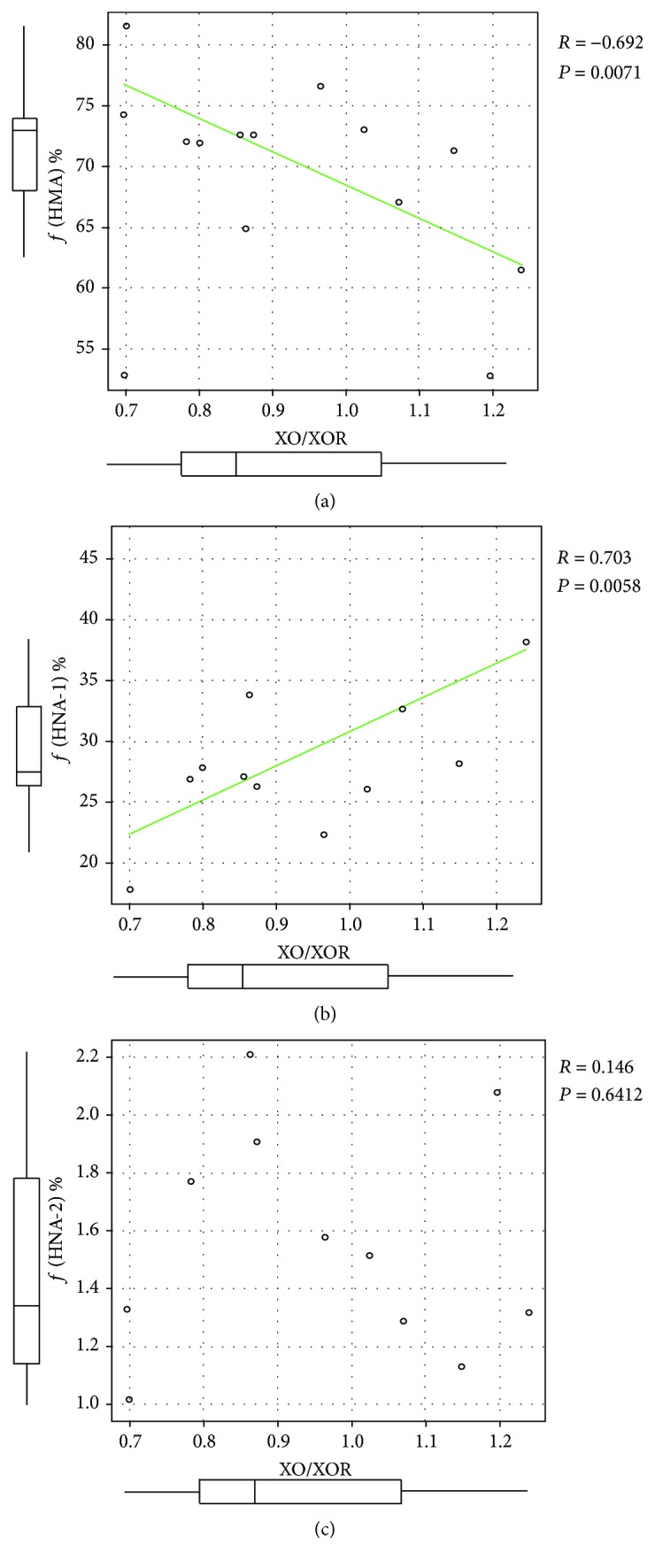
Relationship between XOR redox (XO/XOR) and (a) ƒ(HMA), (b) ƒ(HNA-1), and (c) ƒ(HNA-2). For each value, ƒ(HMA) shows a negative correlation, ƒ(HNA-1) shows a positive correlation, and ƒ(HNA-2) shows a no correlation with XO/XOR.

The relationship between ƒ(HMA), ƒ(HNA-1), ƒ(HNA-2), and XOR redox (XO/XOR) is shown in Figure 3. A significantly negative correlation was observed between XO/XOR and ƒ(HMA) (*R* = −0.692, *P* = 0.0071). In contrast, a significantly positive correlation was observed between XO/XOR and ƒ(HNA-1) (*R* = 0.703, *P* = 0.0058), suggesting that “reversible” oxidation of HSA is closely related to plasma XOR redox. ƒ(HNA-2) showed no apparent correlation with XO/XOR (*R* = 0.146, *P* = 0.6412), indicating that plasma XOR redox is not related to “irreversible” oxidation of HSA.

## 4. Discussion

In this study, we evaluated HSA redox as a marker of OS concurrently with XOR redox. As a result, we found a close relationship between ƒ(HMA), ƒ(HNA-1), and XOR redox. This is the first study to demonstrate that this marker of OS is significantly correlated with the XOR system.

In examining the relationship between HSA redox and kidney function, we found that the reduced albumin fraction (ƒ(HMA)) showed a positive correlation, and oxidized albumin fraction (ƒ(HNA-1) and ƒ(HNA-2)) showed a negative correlation with eGFR. These results are consistent with our previous report [3]; thus, guaranteeing correctness with regard to selection of participants and handling of plasma specimens.

XOR is known as a key enzyme of urate synthesis, which catalyzes the oxidation of hypoxanthine to xanthine and further catalyzes the oxidation of xanthine to the final product uric acid. The molecular weight of XOR is about 300 kDa, which is far larger than that of HSA (68 kDa). Almost all creatures, including animals, plants, fungi, and bacteria, produce XOR as house-keeping enzyme. In creatures without mammals, XOR is found only as XDH form, which utilizes nicotinamide adenine dinucleotide (NAD^+^) as an electron acceptor and does not produce reactive oxygen species (ROS), while in mammals, XOR is found not only as XDH form but also as XO form, which utilizes oxygen molecule as an electron acceptor and generates ROS such as superoxide anion radicals and hydrogen peroxide [12]. XDH and XO are single-gene products [13] and XDH→XO conversion is caused by disulfide compounds via reversible or nonreversible oxidation of the thiol group in XDH [14].

In human, XOR is distributed mainly to the liver, the kidney, the small intestine, and the vascular endothelium. XO/XOR ratio of the capillary endothelium is reflected by plasma XO/XOR ratio [15]. Therefore, theoretically, an increase in the plasma XO/XOR ratio could accelerate vascular OS of via ROS generation and could impair endothelial functions.

Regarding the relationship between HSA redox and XOR redox, a lower XO/XOR ratio was closely related to higher ƒ(HMA); HSA has an intact mercapto group. Because a higher ƒ(HMA) or HMA concentration contributes to lower CVD incidence and mortality burden among CKD patients treated with maintenance dialysis therapy [4], a strategy for suppressing XO/XOR may decrease CVD risk among CKD patients.

While the HNA-1-HSA fraction, which has a mercapto group that can be reversibly oxidized, showed significant positive relationship with XO/XOR, the fraction of HNA-2-HSA, which has a mercapto group that is irreversibly oxidized, showed no relationship with XOR redox. In vivo, HNA-1 is thought to be converted (reduced) to HMA continuously by vascular endothelial cells [16]. A potential influence of endothelial XOR conditions on HNA-1→HMA conversion by endothelial cell has been suggested, as plasma XO/XOR reflects the XO/XOR of the capillary endothelium [15]. In contrast to HNA-1, HNA-2 is not reduced by endothelial cells [16]: the ƒ(HNA-2) level is correlated with serum protein carbonyl formation, an “irreversible” protein modification [17]. The present results suggest that the endothelial XOR condition has a minimal or no influence on irreversible HSA oxidation.

HSA is the most important extracellular antioxidant: more than 70% of the free radical-trapping activity of serum is owing to HSA [18]. Recent article of *Oxidative Medicine and Cellular Longevity* reported that depletion in the efficacy of total plasma antioxidant is significantly related to CVD risk factors [19]. On the other hand, administration of the XOR inhibitor allopurinol reduced CVD risk among a CKD cohort to approximately 30% [7]. To sum up these findings and considering the close relationship between OS and future CVD risk among advanced CKD patients [4, 16], the XOR inhibitor may reduce the endothelial XO/XOR ratio and increase ƒ(HMA); however, further studies are needed to confirm this.

## 5. Conclusion

We measured HSA redox as a marker of OS concurrently with XOR redox in CKD patients and found a close relationship between XOR redox and OS. Strategies for suppressing XO/XOR may decrease the CVD risk of CKD patients via OS reduction.

## Figures and Tables

**Table 1:** Patient characteristics.

Age (years)	62.1 ± 18.8
Gender (% male)	53.8
BMI (kg/m^2^)	24.2 ± 4.3
Blood pressure (mmHg)	
Systolic	136 ± 22
Diastolic	84 ± 6
Primary CKD *n*	
Chronic glomerulonephritis	9
Nephrosclerosis	2
Polycystic kidney	1
Others	1
History of cardiovascular disease (%)	7.7
Antihypertensive medication (%)	84.6
Usage of erythrocyte-stimulating factor (%)	15.4
Usage of xanthine-oxidoreductase inhibitor (%)	61.5

**Table 2:** Clinical data including XOR and HSA redox.

Serum creatinine (mg/dL)	1.68 ± 1.14
Estimated glomerular filtration rate (mL/min/1.73 m^2^)	42.7 ± 23.4
C-reactive protein (mg/dL)	0.14 ± 0.17
Serum uric acid (mg/dL)	5.6 ± 1.9
XO/XOR	0.940 ± 0.183
HSA redox (%)	
ƒ(HMA)	70.16 ± 7.27
ƒ(HNA-1)	28.37 ± 7.06
ƒ(HNA-2)	1.48 ± 0.41

XO: xanthine oxidase; XOR: xanthine oxidoreductase; HSA: human serum albumin; HMA: human mercaptoalbumin; HNA-1: human nonmercaptoalbumin-1; HNA-2: human nonmercaptoalbumin-2.

## Data Availability

The data used to support the findings of this study are available from the corresponding author upon request.
